# Increased understanding of complex neuronal circuits in the cerebellar cortex

**DOI:** 10.3389/fncel.2024.1487362

**Published:** 2024-10-21

**Authors:** Soyoung Jun, Heeyoun Park, Muwoong Kim, Seulgi Kang, Taehyeong Kim, Daun Kim, Yukio Yamamoto, Keiko Tanaka-Yamamoto

**Affiliations:** ^1^Brain Science Institute, Korea Institute of Science and Technology (KIST), Seoul, Republic of Korea; ^2^Division of Bio-Medical Science and Technology, KIST School, Korea University of Science and Technology (UST), Seoul, Republic of Korea; ^3^Department of Integrated Biomedical and Life Sciences, Korea University, Seoul, Republic of Korea; ^4^Department of Life Science, Korea University, Seoul, Republic of Korea

**Keywords:** cerebellar cortex, neuronal circuits, heterogeneity, Purkinje cells, granule cells, molecular layer interneurons

## Abstract

The prevailing belief has been that the fundamental structures of cerebellar neuronal circuits, consisting of a few major neuron types, are simple and well understood. Given that the cerebellum has long been known to be crucial for motor behaviors, these simple yet organized circuit structures seemed beneficial for theoretical studies proposing neural mechanisms underlying cerebellar motor functions and learning. On the other hand, experimental studies using advanced techniques have revealed numerous structural properties that were not traditionally defined. These include subdivided neuronal types and their circuit structures, feedback pathways from output Purkinje cells, and the multidimensional organization of neuronal interactions. With the recent recognition of the cerebellar involvement in non-motor functions, it is possible that these newly identified structural properties, which are potentially capable of generating greater complexity than previously recognized, are associated with increased information capacity. This, in turn, could contribute to the wide range of cerebellar functions. However, it remains largely unknown how such structural properties contribute to cerebellar neural computations through the regulation of neuronal activity or synaptic transmissions. To promote further research into cerebellar circuit structures and their functional significance, we aim to summarize the newly identified structural properties of the cerebellar cortex and discuss future research directions concerning cerebellar circuit structures and their potential functions.

## 1 Introduction

Our understanding of the cerebellar circuit structures has gradually evolved, as numerous studies have clarified previously unknown synaptic connections, organization of circuit formation, or cell types. These structural properties appear to make cerebellar circuits more complex than previously understood, potentially serving as effective components for the expansion of neuronal signals transmitted through the cerebellum. In this article, we summarize these structural updates and discuss their possible contributions to neural computations. The structural updates introduced here are obtained from studies mainly in rodents unless otherwise stated. Although our focus is on structural properties, we also describe functionally identified properties related to the updated structures. Furthermore, based on our revised understanding, we consider the future direction of research related to new structural properties of cerebellar circuits.

Before discussing recent updates, it is important to summarize the fundamental structures of the cerebellum. At the gross morphology level, the cerebellar cortex comprises three tightly folded layers: the granular layer (GL), Purkinje cell layer (PCL), and molecular layer (ML) from the inside. The basic circuit structures within the cerebellum and the morphology of individual types of neurons have long been known (Eccles et al., 1967; Ito, 2006). There are two major afferent pathways into the cerebellum, climbing fibers (CFs) and mossy fibers (MFs). While CFs directly innervate Purkinje cells (PCs), MFs indirectly innervate PCs via granule cells (GCs), which have somas and short dendrites in the GL and their parallel fiber (PF) axons in the ML. PCs provide the sole output from the cerebellar cortex, projecting mainly to the cerebellar nuclei (CN) and vestibular nucleus. PC somas are aligned in the PCL, and their highly elaborate dendritic trees are located in the ML, arranged in a single sagittal plane. This morphology of PC dendrites is beneficial for receiving inputs from many PFs, which run parallel to the layer structures and intersect vertically with PC dendrites. Conversely, a single PC receives synaptic inputs from only one CF, although hundreds of synapses form between them. Molecular layer interneurons (MLIs) receive inputs from PFs and inhibit PCs. Another type of inhibitory interneurons, Golgi cells (GoCs), receive inputs from both PFs and MFs, and inhibit GCs. These typical cerebellar circuit structures are strikingly conserved throughout the cerebellum and are believed to be crucial for cerebellar neural computations.

## 2 Update of the structural organization of GCs

**Figure 1:**
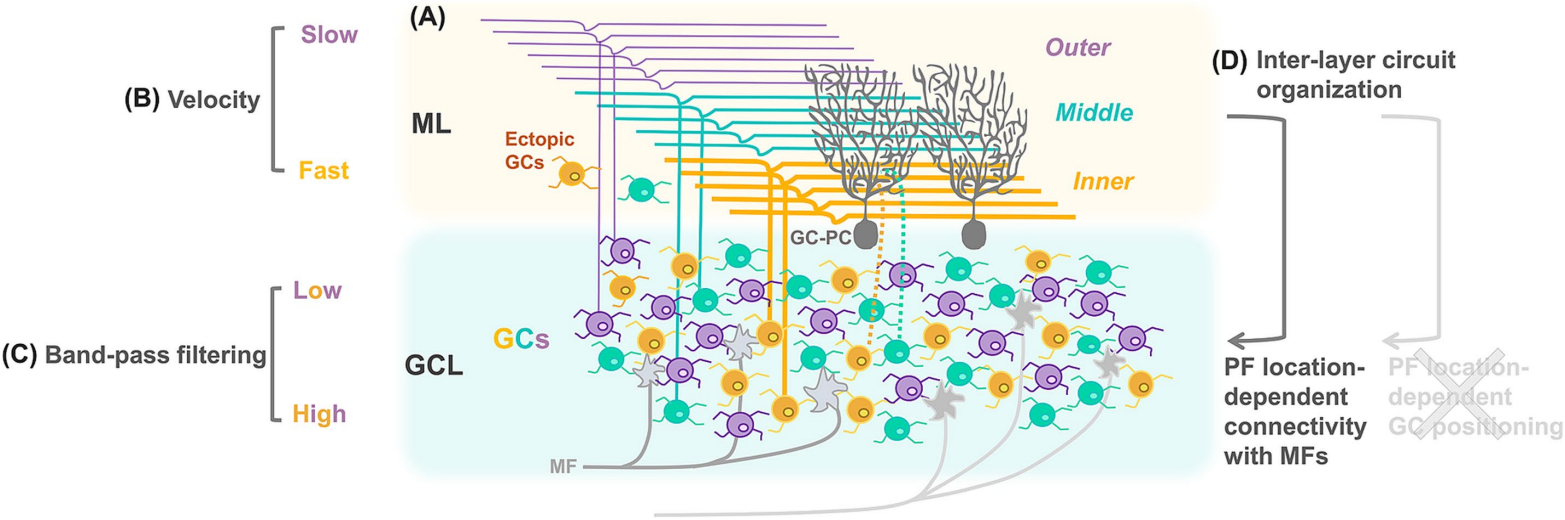
Schematic diagram showing heterogeneous properties of cerebellar GCs. PF diameters **(A)** and the velocity of action potential propagation **(B)** are varied according to the location of PFs in the ML. Firing properties of GCs are varied according to their own locations in the GL **(C)**. Whereas distributions of GCs and PFs appear not to be spatially correlated, connectivity of GCs with MFs in the GL is organized according to the location of their PFs in the ML **(D)**.

Cerebellar GCs are the most abundant neurons in the brain, consisting of more than 50% of all brain neurons (Herculano-Houzel et al., 2015). GCs have a unique morphology, characterized by a very small soma with only 3–4 short dendrites, and an ascending axon extending into the ML, where bifurcated PFs are formed. Each of the 3–4 dendrites of a GC makes a synapse with a single MF terminal in the glomerulus. Although these numerous small GCs have simply been considered homogeneous, their heterogeneous properties have also been discovered in terms of molecular expression (Lein et al., 2007; Kozareva et al., 2021), morphology (Houston et al., 2017), and physiological properties (D'Angelo et al., 1998; Gandolfi et al., 2014; Chabrol et al., 2015; Dorgans et al., 2019; Masoli et al., 2020; Straub et al., 2020; Rhee et al., 2021; Shuster et al., 2021). A well-known anatomical heterogeneity is the position of individual PFs within the ML, resulting in the formation of characteristic laminar structures by all PFs (Figure 1A). PF heterogeneity is evident not only in their position but also in their varying diameters, i.e., they are thinner in the outer ML and thicker in the inner ML (Sultan, 2000; Wyatt et al., 2005; Straub et al., 2020). Consistent with the general correlation between axon diameter and action potential propagation velocity (Schmidt and Knösche, 2019), the velocity is higher in the inner layer (Figure 1B; Straub et al., 2020). Differences also exist in the firing properties of GCs within the inner and outer GL (Figure 1C; Straub et al., 2020), suggesting that these distinct firing properties might be transmitted to spatially correlated PFs in the ML. However, several studies have demonstrated no spatial correlation between GC somas in the GL and PFs in the ML (Zong et al., 2005; Wilms and Häusser, 2015; Markwalter et al., 2019; Rhee et al., 2021; Kim et al., 2023b), indicating that the properties of GCs detected in the GL cannot be directly related to the locations of PFs in the ML. Advanced techniques linking specific GCs to their PF locations are required to characterize GCs in the GL according to PF locations. One study used adeno-associated virus (AAV)-mediated labeling of a group of GCs with a bundle of PFs in the ML and demonstrated MF stimulation-mediated, nonuniform calcium responses in GCs specific to their PF locations (Rhee et al., 2021). Additionally, GCs with PFs in different ML sublayers exhibit moderately, yet significantly, different connectivity with MFs of varying origins (Figure 1D; Shuster et al., 2021; Kim et al., 2023b). Thus, PF location-dependent variability in GC properties, namely, action potential propagation velocity in PFs, functional properties in GCs, and connectivity with MFs, has gradually been revealed.

It would be interesting to clarify the role of these GC properties according to PF locations. In general, a conceptual role of GCs is considered to be separation of contextual information (Cayco-Gajic and Silver, 2019). Characteristic GC circuit properties, such as large expansion and sparse synaptic connectivity from MFs to GCs, and broad feedback inhibition mediated by GoCs, are advantageous for pattern separation (Billings et al., 2014; Cayco-Gajic et al., 2017; Litwin-Kumar et al., 2017). One traditional theory for pattern separation is sparse coding, which posits that only sparse populations of GCs are activated to convey specific types of information. This theory can explain the associative learning that occurs at synapses with PC dendrites (Marr, 1969; James, 1971). However, *in vivo* calcium imaging studies suggested that activities in denser-than-expected GC populations represent specific type of contextual information (Giovannucci et al., 2017; Knogler et al., 2017; Wagner et al., 2017). In contrast, isolated sensory stimuli were recently reported to trigger relatively sparse GC population responses via local synaptic inhibition (Fleming et al., 2024). Computational studies suggested that the sparse synaptic connections between MFs and GCs may contribute to the pattern separation of contextual information, regardless of whether GC population activity is sparse or dense (Cayco-Gajic et al., 2017). It is not entirely clear whether GCs enhance pattern separation via sparse coding or other computational mechanisms. In any case, an increased representation of neuronal signals appears beneficial for pattern separation, which may be achieved through the combination of PF location-dependent GC properties described in the previous paragraph.

In addition to the PF location-dependent organization of GCs, other GC-related structural updates have been reported. The analysis of large-scale electron microscopy data revealed structured connectivity not only from MFs to GCs but also from GCs to PCs (Nguyen et al., 2023). Compared with randomized connections, pairs of GCs tended to share MF inputs, and pairs of PCs exhibited similar PF input patterns. A computational model predicted that such structured connectivity increased resilience to noise without significantly affecting pattern separation capacity. In another study, ectopic GCs located in the ML, long thought to have simply ceased migration during development, were found to be more abundant in the posterior cerebellum than previously expected—comprising approximately 40% of the population relative to MLIs—and exhibited firing properties similar to those of GCs located in the GL (Dey et al., 2022). This raises the possibility that ectopic GCs may have a unique functional role.

## 3 Update of the circuit surrounding MLIs

MLIs, a major type of inhibitory interneuron in the cerebellum, provide feedforward inhibition to PCs by receiving excitatory synaptic inputs from PFs. They also receive inputs from neighboring MLIs through chemical and/or electrical synapses (Häusser and Clark, 1997; Mittmann et al., 2005; Rieubland et al., 2014). MLIs are traditionally classified into two cell types: basket cells (BCs) and stellate cells (SCs), based on several anatomical characteristics (Eccles et al., 1967; Jörntell et al., 2010). BCs, with relatively straight dendrites, are mainly located in the inner one-third of the ML, while SCs, with dendrites and axons within a more limited area, are mainly located in the outer two-thirds of the ML (Schilling, 2013; Sotelo, 2015; Sergaki et al., 2017; Kim and Augustine, 2021). Notably, BCs innervate the area around the PC somas, and form pericellular baskets on the PC somas and dense plexuses called pinceau on the PC axon initial segment (AIS), providing strong inhibitory control of PC activity (King et al., 1993; Sultan and Bower, 1998; Bower, 2010; Sotelo, 2015). In contrast, SCs send inhibitory inputs to PC dendrites (Sotelo, 2015; Sergaki et al., 2017). Beyond these basic anatomical properties, higher-order circuit mechanisms also contribute to MLI functions. As the characteristics of MLIs have been comprehensively summarized in other review articles (e.g., Jörntell et al., 2010; Sotelo, 2015; Kim and Augustine, 2021), we focus on new information associated with MLI circuits and discuss their possible organization.

### 3.1 Cell types of MLIs

**Figure 2:**
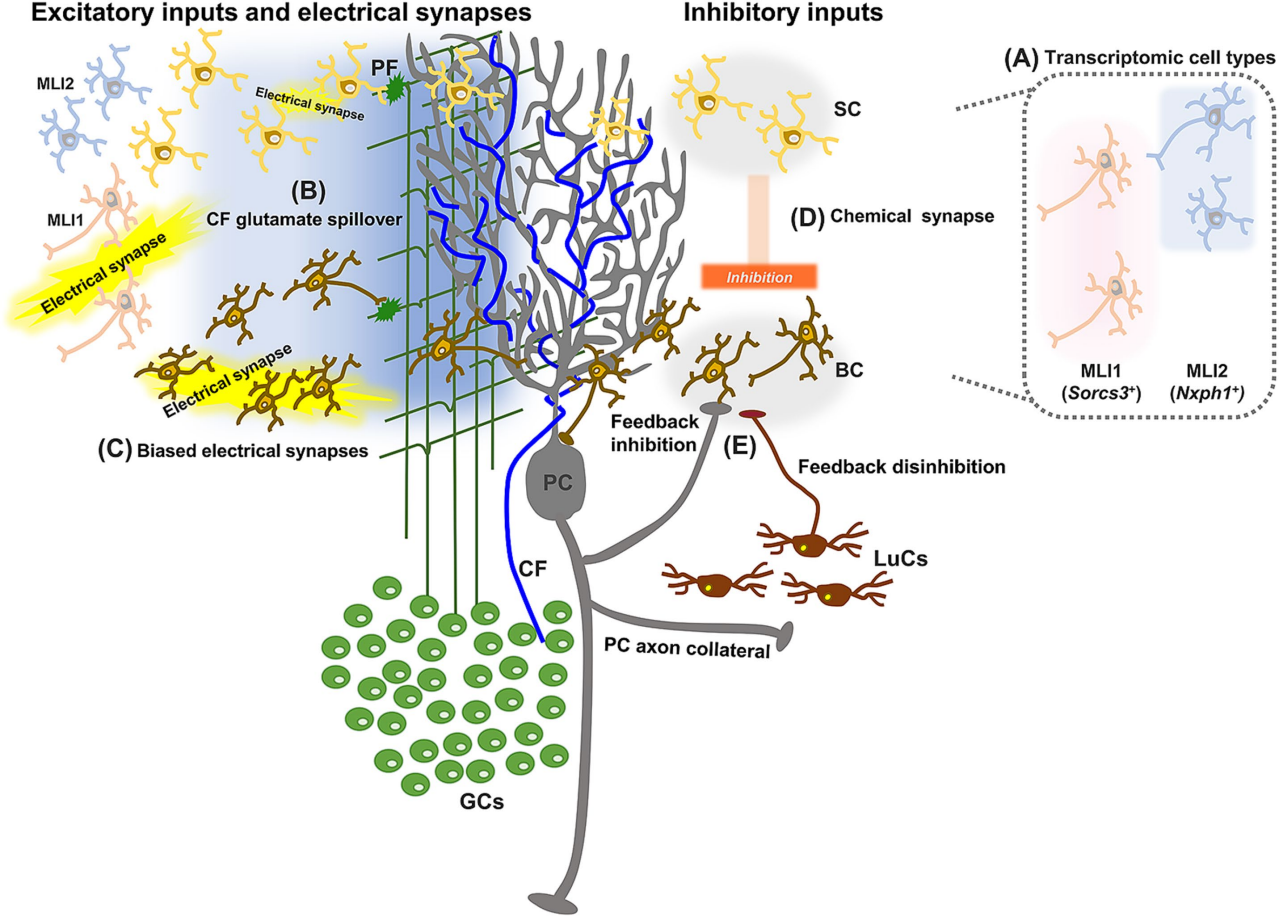
Schematic diagram showing update of the neuronal circuits surrounding MLIs. In addition to well-established circuit structures, studies revealed two distinct types of MLIs based on molecular expression **(A)**, spillover-mediated CF-MLI connections **(B)**, organized electrical coupling between MLIs **(C)**, MLI-MLI synaptic connections biased from the outer to the inner ML **(D)**, and inhibitory inputs from PCs to MLIs **(E)**.

Even though BCs and SCs are the two stereotyped MLIs, the classification of MLIs is still under debate. There is a contrasting idea to the traditional classification of BCs and SCs; all MLIs originate from a single type of interneurons with continuous variation in morphology and different target locations on PCs (Rakic, 1972; Sultan and Bower, 1998; Bower, 2010). A transcriptome study identified two distinct types of MLIs, MLI1s and MLI2s, characterized by different molecular expressions, such as *Sorcs3* in MLI1s and *Nxph1* in MLI2s (Figure 2A; Kozareva et al., 2021). Because both MLI1s and MLI2s showed similar continuum in morphological properties, particularly an SC-like morphology in the outer one-third of the ML, they do not align with the traditional BC and SC classification. However, further analyses revealed at least two distinct morphological aspects between MLI1s and MLI2s (Lackey et al., 2024). First, MLI1 somas are spiny, while MLI2 somas are smooth. Second, MLI1s in the inner two-third of the ML contribute to pinceau formations, whereas all MLI2s, regardless of soma location, retain classical SC morphologies without contributing to pinceau. Supporting this second observation, another study identified two discrete morphological types of MLIs, canonical BCs and SCs with extensive heterogeneity, which are distinguished by the morphological features of axons rather than their soma positions in the ML (Wang and Lefebvre, 2022). This study confirmed the dissociations between morphological and transcriptomic types, but showed some relationships between them. *Sorcs3*-positive MLIs, namely MLI1s, are mostly BCs and SCs with long axons, further distinguished by the combined expression levels of *Grm8* and *Cacna1e*. *Nxph1*-positive MLIs, namely MLI2s, are largely SCs with short axons and partly SCs with long axons located in the superficial layer.

In general, neuronal cell-type classification considers not only transcriptional and morphological properties, but also electrophysiological and connectional properties (Zeng and Sanes, 2017). MLI2s were found to be more excitable than MLI1s, and the expression of *Gjd2*, the gene encoding the dominant gap junction protein, connexin 36, was detected in MLI1s, but not in MLI2s (Kozareva et al., 2021), indicating electrical differences in the two transcriptionally identified cell types. In terms of connectional properties, a subpopulation of MLIs located in the inner one-third of the ML was found to receive inhibitory inputs from PCs (Witter et al., 2016; Halverson et al., 2022), which may contribute to the disinhibition of other PCs. Further studies are required to clarify whether these MLIs have other characteristic properties and can be categorized into a specific cell type based on the functional uniqueness.

The anatomical organization of MLIs and their circuits, which affect cell type classification, appears to rely on process occurring during the postnatal developmental period. MLIs are gradually generated from precursors in the white matter (WM), which originate in the ventricular zone (Yamanaka et al., 2004). Immature postmitotic MLIs radially migrate toward the ML, where they find their final positions through a complex migratory route involving radial and tangential migration (Simat et al., 2007a; Cameron et al., 2009; Park et al., 2021). This complex migration is regulated by several pathways, such as excitatory and inhibitory synaptic transmission (Wefers et al., 2017). Specifically, the blockade of synaptic transmission from PF bundles resulted in abnormal distributions of MLIs (Park et al., 2019), indicating that PF inputs likely regulate the migration that determines the final positions of MLIs. The final position of MLIs also depends on their timing of birth; early-and late-born MLIs are positioned in the inner and outer parts of the ML, respectively, meaning that BCs are generally born earlier than SCs (Yamanaka et al., 2004; Weisheit et al., 2006; Leto et al., 2008; Schilling et al., 2008; Sudarov et al., 2011; Consalez and Hawkes, 2012; Sotelo, 2015), although their positions are not strictly defined. Despite differences in morphology, transcriptional and electrophysiological properties, or connectivity in mature MLIs, MLI precursors before leaving the WM are indistinguishable in terms of their genetic markers (Maricich and Herrup, 1999; Leto et al., 2006; Carter et al., 2018). This suggests that each MLI acquires its characteristic phenotypes after migration begins. It has been proposed that an additional tangential migration, supported by premigratory GCs in the EGL, is required for the synaptic differentiation of SCs (Cadilhac et al., 2021). Additionally, a study linking morphological and transcriptomic types demonstrated that BC and SC morphological identities diverged during early phases of migration, prior to the expression of transcriptomic markers (Wang and Lefebvre, 2022). Thus, the diverse characteristics of MLIs may arise from the impact of their migration process itself or the environmental situations encountered during migration.

### 3.2 Excitatory inputs to MLIs

As described above, PFs are well-known excitatory inputs to MLIs. With the revelation of the heterogeneous properties of GCs and PFs, different patterns of short-term synaptic plasticity (STP) were observed in PF synapses of the two types of MLIs, namely, BCs and SCs. The PF-SC synapses demonstrated persistent facilitation, whereas PF-BC synapses showed depression following initial paired-pulse facilitation (Bao et al., 2010). This implies that PF inputs trigger firing in BCs first, followed by delayed firing in SCs. Given their target locations on PCs, PF inputs may sequentially hyperpolarize PCs from soma to dendrites (Bao et al., 2010; Blackman et al., 2013). A recent study further categorized STP in unitary PF-MLI synapses into four types, attributing this diversity to the heterogeneous expression of the key STP molecule, synapsin II, in GCs, but found no clear MLI type-dependent STP patterns (Dorgans et al., 2019). Although it is unclear how to reconcile these conflicting results in terms of cell types, the diversity of PF-MLI synaptic functions may contribute to increased coding patterns, potentially allowing efficient separation of different contexts in the cerebellum.

In contrast to the clear excitatory synaptic connections from PFs to MLIs, excitatory inputs from CFs to MLIs have long remained controversial. Although anatomical studies of labeled CFs suggested possible synaptic connections between CFs and MLIs (Palay and Chan-Palay, 1974; Sugihara et al., 1999), no conventional synaptic architecture was found between them in electron microscopy studies (Hámori and Szentágothai, 1980; Kollo et al., 2006). Nevertheless, functional effects of CFs were detected in MLIs (Eccles et al., 1966; Jörntell and Ekerot, 2003). It became clear that CF-MLI connections were mediated uniquely, through glutamate spillover (Figure 2B; Szapiro and Barbour, 2007; Mathews et al., 2012; Coddington et al., 2013). CF synapses to PCs have a very high release probability and typically show multivesicular release (Wadiche and Jahr, 2001; Foster et al., 2002; Harrison and Jahr, 2003), so that CF activation may reasonably lead to glutamate spillover. The involvement of N-methyl-D aspartate (NMDA) receptors in the spillover-mediated CF-MLI connections is also plausible, considering that MLIs have NMDA receptors on their dendrites (Carter and Regehr, 2000; Clark and Cull-Candy, 2002). Thus, it is now generally accepted that MLIs receive CF inputs via glutamate spillover. This spillover-mediated, CF-dependent regulation of MLIs has been shown to be involved in sensory processing *in vivo* (Arlt and Häusser, 2020).

### 3.3 Electrical synapses between MLIs

As commonly seen in inhibitory interneurons of the mammalian brain (Galarreta and Hestrin, 2001; Pereda, 2014; Coulon and Landisman, 2017), MLIs are also connected with each other via electrical synapses (Mann-Metzer and Yarom, 1999). The electrical synapses between MLIs rely on the gap junction protein connexin 36, which is differentially expressed according to their locations in the ML (Alcami and Marty, 2013). MLIs in the inner ML, presumably BCs, have higher levels of connexin 36 than those in the outer ML. Consistently, electrophysiological recordings have demonstrated that electrical connections are more frequent and stronger in BCs than in SCs (Figure 2C; Alcami and Marty, 2013). The difference in electrical connections is clear between MLI1s and MLI2s, as the expression of *Gjd2* and electrical coupling were found only in MLI1s, but not ML2s (Kozareva et al., 2021; Lackey et al., 2024). Given that BCs are likely part of MLI1s (Wang and Lefebvre, 2022; Lackey et al., 2024), these results regarding cell type-dependent electrical connections appear consistent. Furthermore, studies using multiple patch-clamp recordings and optogenetic mapping identified that electrical coupling occurs in the sagittal plane (Kim et al., 2014; Rieubland et al., 2014). Such structured organization of electrical connections between MLIs contributes to the regulation of feedforward inhibition of PCs, by generating spatiotemporally controlled synchronization of MLI activity (Hoehne et al., 2020).

### 3.4 Inhibitory inputs to MLIs

MLIs are well known to receive GABAergic inhibitory synaptic inputs from neighboring MLIs (Llano and Gerschenfeld, 1993; Häusser and Clark, 1997; Mittmann et al., 2005), and this inhibitory connection is presumably important for fine-tuning the temporal precision of spiking in PCs (Mittmann et al., 2005). Similar to the electrical connections between MLIs, their inhibitory synaptic connections were also found to be oriented in the sagittal plane, although not as strongly biased as electrical coupling (Rieubland et al., 2014). Additionally, transitive patterns of MLI-MLI synaptic connections were demonstrated to be organized from the outer to the inner part of the ML (Figure 2D; Arlt and Häusser, 2020). This raises the possibility that such MLI microcircuit organization, together with the location of activated PFs, may be utilized for the regulation of cerebellar information processing – e.g., additional activation of PFs in the outer ML may lead to greater excitation of PCs through the reduction of MLI-dependent inhibition of PCs. Cell type-dependent innervation patterns have recently been demonstrated: MLI1s mainly innervate PCs to inhibit them, while MLI2s mainly innervate MLI1s to disinhibit PCs (Lackey et al., 2024). In other words, many MLI1s would receive relatively strong inhibitory inputs from MLI2s, whereas MLI2s may have little or no inhibitory inputs from other MLIs.

In addition to the inhibitory synaptic inputs from MLIs, early anatomical studies have suggested other possible inputs from PCs and Lugaro cells (LuCs), the latter of which are a type of inhibitory interneuron in the GL (Hámori and Szentágothai, 1968; Larramendi and Lemkey-Johnston, 1970; O’Donoghue et al., 1989; Lainé and Axelrad, 1996, 1998). While functional synaptic connections from LuCs to MLIs are not yet clarified, functional inhibitory inputs from PCs to MLIs were identified by recording synaptic currents evoked by the optogenetic stimulation of PCs (Figure 2E; Witter et al., 2016; Halverson et al., 2022). Optogenetically evoked synaptic currents were detected in approximately 16 to 30% of MLIs, indicating these are not extremely rare synaptic connections. Reciprocally connected pairs of PCs and MLIs have not been found so far, thus MLIs receiving inputs from PCs are considered to send inhibitory inputs to other PCs, suggesting that this pathway from PCs to MLIs contributes to synchronization of PCs through disinhibition of MLIs (Halverson et al., 2022). Given that inhibitory LuCs also receive inputs from PCs (Witter et al., 2016), PC population activity may rely on the organization of connections among PCs, LuCs, and MLIs, if LuCs functionally inhibit MLIs. Direct connections from PCs to MLIs are considered to mediate synchronization of neighboring PCs (Witter et al., 2016; Halverson et al., 2022), while indirect connections from PCs to MLIs through LuCs may contribute to desynchronization of other PC populations, as seen between aldolase C/zebrin II-positive and-negative PC populations (Tsutsumi et al., 2015). In addition, whereas glutamate spillover from CFs primarily exerts excitatory influence to MLIs, as described above, other MLIs located outside the glutamate diffusion limit have been shown to be inhibited upon CF activation via disynaptic inhibition (Coddington et al., 2013). Thus, CF-mediated functional segregation of MLIs may also contribute to the distinctive patterns of activity of different PC populations. Alternatively, segregation of MLIs could be along the perpendicular direction to the layer, as CF inputs were reported to activate many MLIs but inhibit MLIs in the deep ML that have strong inhibitory impacts onto PCs (Arlt and Häusser, 2020).

## 4 Newly identified circuit properties in PCs

PCs are a unique type of neuron specific to the cerebellar cortex and have been extensively studied from various perspectives, including development, dendritic arborization, synapse formation, electrophysiological properties, synaptic regulation, molecular expression, and neuronal circuits (Yuzaki, 2004; Sotelo and Dusart, 2009; De Zeeuw et al., 2011; Cerminara et al., 2015; Leto et al., 2016; Nedelescu et al., 2018; De Zeeuw, 2021). PCs provide the sole output of the cerebellar cortex and form GABAergic inhibitory synapses with neurons in target regions outside the cerebellar cortex, mainly in the CN. Despite the wealth of knowledge about PCs, properties of PC axon collaterals and ephaptic coupling around PCs had not been adequately analyzed, leaving their importance in cerebellar computations and functions unclear. However, in the past decade, several studies have provided more detailed characterizations of these aspects. Additionally, the morphological diversity and dopaminergic signaling of PCs have also been demonstrated.

### 4.1 PC axon collaterals

**Figure 3:**
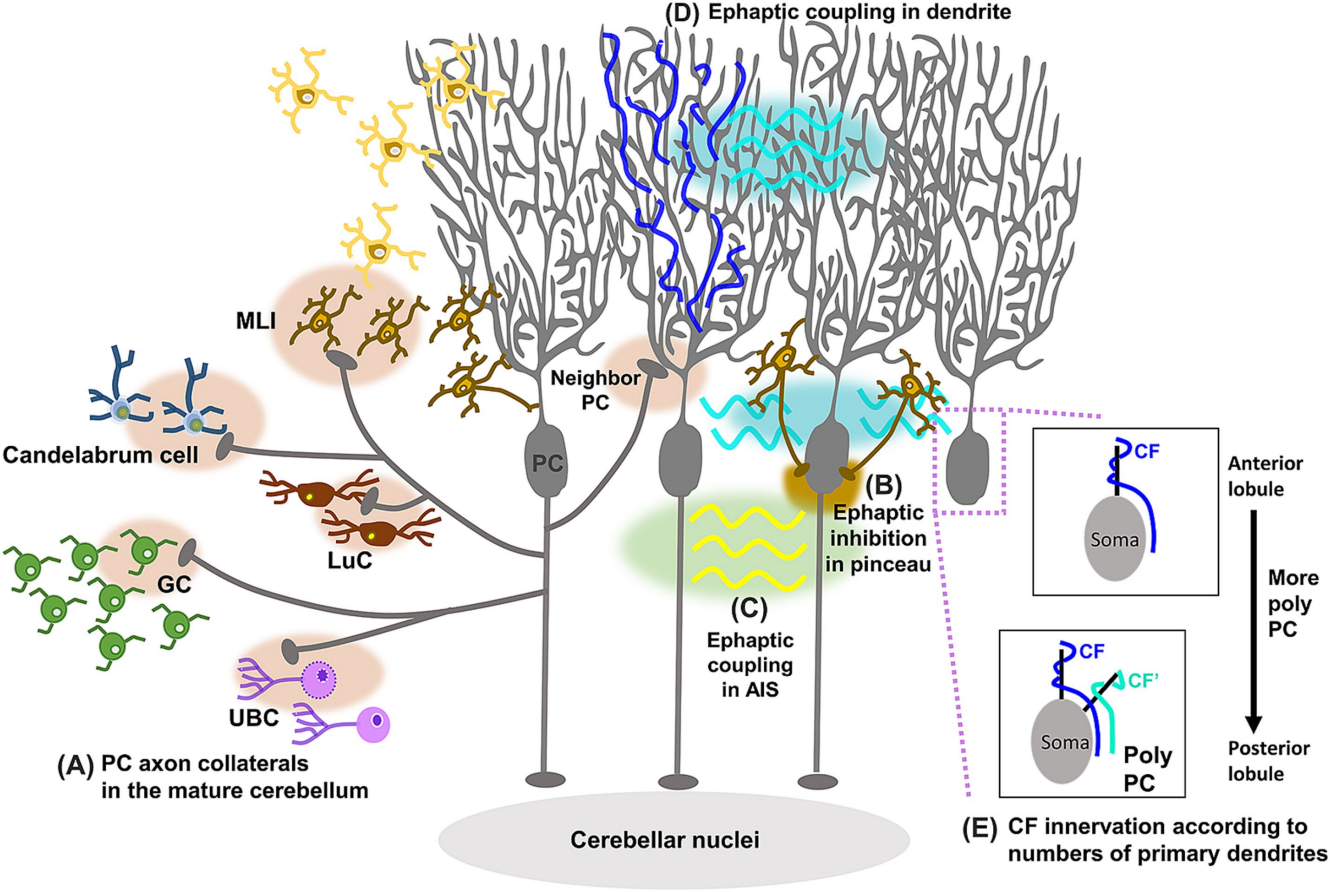
Schematic diagram showing update of PC circuit structures. PC axon collaterals form functional synaptic connections **(A)** to other PCs, MLIs, LuCs, candelabrum cells, GCs, and UBCs in the mature cerebellum. In addition, studies have demonstrated ephaptic coupling between the pinceau in BCs and the AIS in PCs, triggering rapid inhibition of PCs **(B)**, between PCs in the AIS, leading to the synchronization of spontaneous PC firing **(C)**, and between PC dendrites, causing the inhibition of many neighboring PCs upon excitation by CF inputs **(D)**. PCs with two primary dendrites have been found more frequently in posterior lobules, receiving two discrete CF inputs on each of the two primary dendrites **(E)**.

While PC axons project outside the cerebellar cortex, PC axon collaterals enable the modulation of information processing within the cerebellar cortex by sending feedback signals. Early studies already described PC axon collaterals as forming synapses onto other PCs and neurons of the ML (Eccles et al., 1967; Larramendi and Lemkey-Johnston, 1970; Chan-Palay and Palay, 1971). Although they were anatomically observed in both young and adult rodents (Larramendi and Lemkey-Johnston, 1970; Chan-Palay and Palay, 1971; Bishop, 1982; Hawkes and Leclerc, 1989; Bernard et al., 1993), their functional synaptic transmission was first confirmed only in young animals (Orduz and Llano, 2007; Watt et al., 2009; Hirono et al., 2012). These studies demonstrated that PC axon collaterals form functional synapses with other PCs and globular cells, small inhibitory interneurons located in the GL. A computational model and experimental confirmation demonstrated that PC axon collaterals generated waves of PC activity traveling along chains of connected PCs in the developing cerebellum, whereas these waves were not observed in the mature cerebellum (Watt et al., 2009). This led to an idea that PC axon collaterals play a crucial role only in cerebellar circuit formation during postnatal development. In contrast, studies using optogenetic stimulation with patch-clamp recording identified functional synaptic transmissions from PC axon collaterals in adult animals (Figure 3A; Guo et al., 2016; Witter et al., 2016), although these synaptic connections were restricted to the parasagittal plane and were present only near the PCs sending the axon collaterals. GABAergic inhibitory synaptic transmissions were recorded from PCs, MLIs, LuCs, and candelabrum cells (neurons situated in the PCL), but not from GoCs, upon optogenetic stimulation of PCs or spontaneous PC firing (Witter et al., 2016; Halverson et al., 2022; Osorno et al., 2022). While there were variabilities depending on the lobule, PC axon collaterals also projected to the GL, and inhibited GCs and a subset of unipolar brush cells (UBCs) (Guo et al., 2016; Guo et al., 2021). These studies suggest that PC axon collaterals may have additional roles in the mature cerebellum. It would be interesting to differentiate properties of collateral axons in the cerebellar cortex from axons projecting to the CN, as this may lead to techniques to selectively manipulate collateral axons.

### 4.2 Ephaptic coupling around PCs

Signal processing and transmission in the nervous system involve the flow of current across neuronal cell membranes, which alters the electrical potential in extracellular regions. Through these alterations in extracellular potential, neurons can interact with adjacent neurons without forming the specific cell-to-cell contacts required for chemical and electrical synaptic transmission. These electrical interactions, described as early as the 1940s (Katz and Schmitt, 1940; Arvanitaki, 1942), are now called ephaptic transmission or coupling (Anastassiou and Koch, 2015; Faber and Pereda, 2018). In the well-established circuit structures where PFs provide direct excitatory inputs and disynaptic inhibitory inputs through BCs or SCs onto PCs, recordings from PCs during PF volleys were expected to show a sequence of excitation followed by inhibition. However, an initial study revealed that a brief inhibition occurred before this expected sequence (Korn and Axelrad, 1980). Due to its short latency, this brief inhibition was proposed to be electrically generated by nearby BCs. BCs form pinceau that wrap around and terminate on the AIS of PCs (Sotelo, 2015). While BC axons make GABAergic synaptic contacts onto PC somas, the pinceau themselves are largely devoid of chemical and electrical synapses (Ichikawa et al., 2011), raising a question about their functions. The dense plexuses of pinceau appear to be well-suited for generating local electric fields, and indeed, one study demonstrated that the pinceau could induce rapid ephaptic inhibition of PC axons (Figure 3B; Blot and Barbour, 2014). This study proposed that pinceau-dependent ephaptic inhibition allows GCs to instantaneously inhibit PCs. Given the variability in pinceau size (Zhou et al., 2020), this inhibitory regulation may differ depending on PC zones identified by specific molecular markers, such as aldolase C/zebrin II (Apps and Hawkes, 2009; Cerminara et al., 2015).

In addition to the ephaptic coupling between the pinceau in BCs and the AIS in PCs, two other types of ephaptic coupling between PCs have been identified (Han et al., 2018; Han et al., 2020). One type of ephaptic coupling observed in the AIS of PCs led to the synchronization of spontaneous PC firing (Figure 3C; Han et al., 2018). Another type of coupling, occurring between PC dendrites, resulted in the inhibition of multiple neighboring PCs when synaptically connected PCs were activated by CF inputs (Figure 3D; Han et al., 2020). This study proposed that this type of ephaptic coupling allows CF inputs to efficiently activate CN neurons through the simultaneous inhibition of many PCs. Although these two types of ephaptic coupling between PCs may seem contradictory—one causing excitation and the other inhibition of neighboring PCs—they likely contribute to the spatially distinct responses of neighboring PCs upon CF inputs.

### 4.3 PC morphological diversity

The diversity of PCs has been well characterized by different expression levels of marker molecules, such as aldolase C/zebrin II (Cerminara et al., 2015). The expression patterns of these molecules form longitudinally striped compartments known as microzones. PCs in different microzones (e.g., AldoC+ and AldoC-microzones) exhibit distinct firing patterns and project to specific sets of CN or vestibular nuclei, linking each microzone to distinct functions (De Zeeuw, 2021). For instance, fast and slow eye movements are mediated by distinct PC subpopulations in the flocculus, known as 9- and 9+ PCs, respectively (Blot et al., 2023). Apart from these modules visualized by molecular markers, the diversity of PC morphology has been gradually recognized. PCs typically have a characteristic morphology with a large, planar, highly branched dendritic tree, and the diversity is observed in the dendritic shapes (Nedelescu and Abdelhack, 2013; Nedelescu et al., 2018; Chang et al., 2020; Busch and Hansel, 2023; Magnus et al., 2023). In zebrafish, four types of PCs with different morphological and firing properties were classified, and they were active at different phases during swimming, suggesting that different types of PCs have different functions (Chang et al., 2020). In mice, morphological types were specifically considered based on the number of primary dendrites (Nedelescu and Abdelhack, 2013; Nedelescu et al., 2018; Busch and Hansel, 2023). Most PCs in the anterior and central lobules have single primary dendrites, but some PCs, particularly those in the posterior lobules, have two primary dendrites. Furthermore, a recent study (Busch and Hansel, 2023) demonstrated that PCs with two or multiple primary dendrites, called as poly PCs, are a near-universal morphological feature in humans and are functionally different from normative PCs in terms of the number of CF innervations. While PCs usually receive inputs from a single CF, each of the two primary dendrites of poly PCs receives discrete CF inputs (Figure 3E), suggesting that poly PCs could integrate different information from independent CFs. Since poly PCs are more prevalent in the posterior lobules, this information integration would be more beneficial for functions involving the posterior lobules, such as fear-evoked freezing behavior (Koutsikou et al., 2014).

Updates have also been made to our understanding of PC axonal morphology. Axonal swellings, known as torpedoes, were historically associated with pathological conditions (Louis et al., 2009; Babij et al., 2013; Louis et al., 2014; Redondo et al., 2015). However, axonal swellings have also been observed in healthy young animals and are thought to serve a different function from those associated with disease (Ljungberg et al., 2016). In fact, axonal swellings in healthy animals have been shown to enhance action potential fidelity and are linked to cerebellum-dependent motor leaning (Lang-Ouellette et al., 2021). Thus, these swellings appear to represent a form of axonal structural plasticity.

### 4.4 Dopaminergic signaling related to PCs

In addition to influences from traditional synaptic inputs, axon collaterals of other PCs, and ephaptic coupling, PCs have been reported to be affected by dopamine through D2 receptors (Cutando et al., 2022). D2 receptor-mediated dopaminergic signaling appears to play a role in regulating social behaviors. Dopamine release may originate from dopaminergic projection neurons outside the cerebellum, as early studies showed sparse dopaminergic innervation in the cerebellum (Panagopoulos et al., 1991; Ikai et al., 1992; Nelson et al., 1997). Another potential source could be a subset of PCs that express tyrosine hydroxylase, the rate-limiting enzyme in the classical dopamine synthesis pathway (Takada et al., 1993; Abbott et al., 1996). However, a recent study demonstrated that dopamine is synthesized in PCs via an alternative pathway involving a member of the cytochrome P450 superfamily, CYP2Ds (Li et al., 2023). This study also revealed a role for D1 receptors in Bergmann glial cells, contributing to motor and social behaviors. Understanding how dopamine released from PCs acts in a coordinated manner on D2 receptors in PCs and D1 receptors in Bergmann glial cells would be an intriguing avenue for future research.

## 5 Update of the GoC circuits

**Figure 4:**
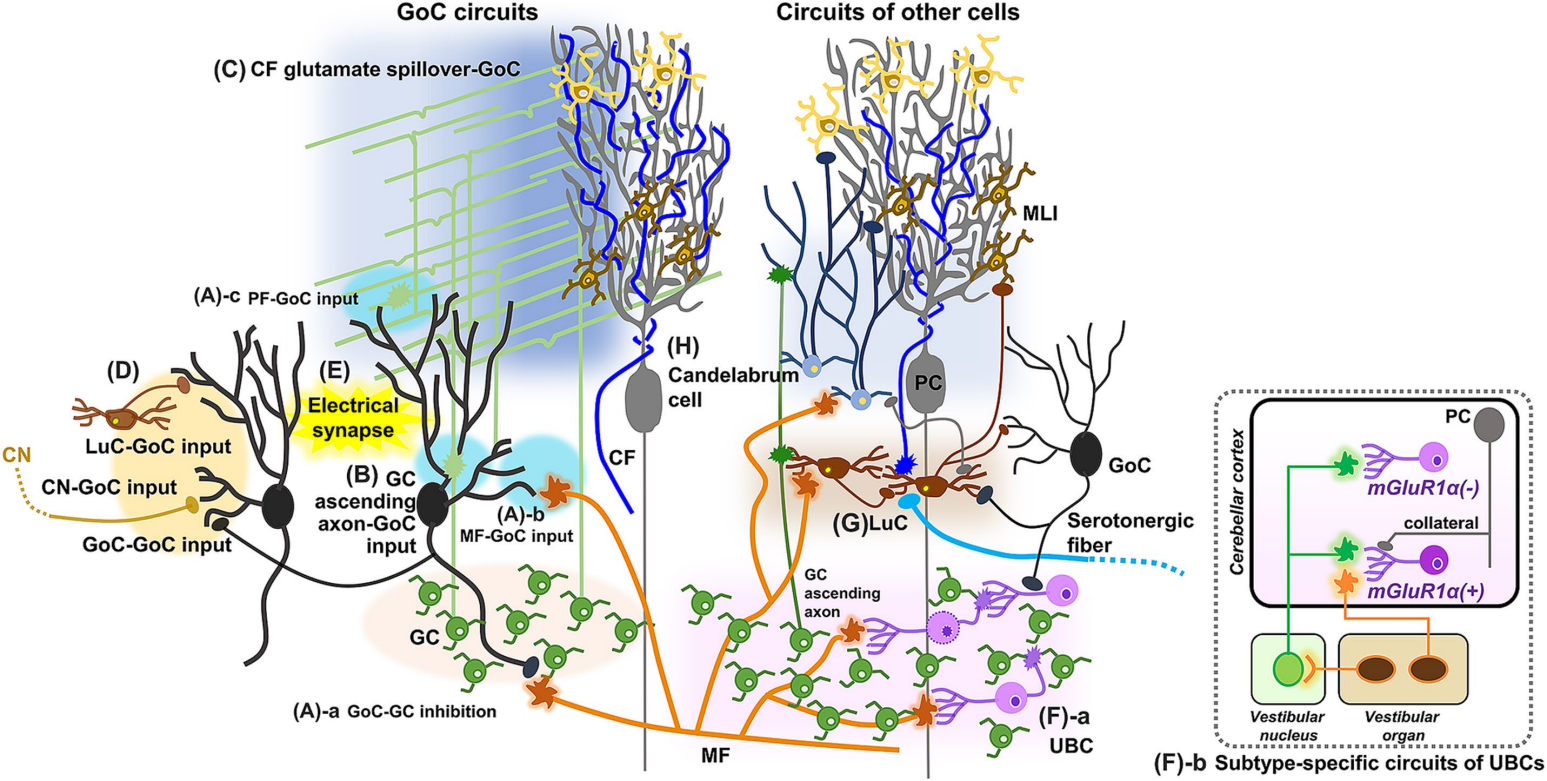
Schematic diagram showing update of GoCs and other cell types. In addition to well-established circuit structures **(A)**, GoCs receive inputs from GC ascending axons **(B)** and spillover-mediated excitatory inputs from the CFs **(C)**, may receive inhibitory inputs from LuCs, other GoCs and CN neurons **(D)**, and have electrical coupling between GoCs **(E)**. UBCs receive excitatory input from a single MF and inhibitory inputs from GoCs, and make synapses onto GCs or other UBCs **(F-a)**. Cell-type specific circuit structures of UBCs are also revealed **(F-b)**. LuCs, projecting to MLIs and probably to GoCs, have diverse inputs from MFs, CFs, GCs, other LuCs, GoCs, PCs, and serotonergic fibers **(G)**. Candelabrum cells receive excitatory inputs from MFs and GCs, and innervate MLIs **(H)**.

Even though GoCs are the most abundant type of inhibitory interneuron in the GL, they are sparse, existing at a ratio of one to several hundred or thousand GCs (D'Angelo and Casali, 2012). These few GoCs are heterogeneous, in terms of their molecular markers, morphology, and electrophysiological properties (Dieudonné, 1998; Geurts et al., 2001; Simat et al., 2007b; Barmack and Yakhnitsa, 2008; D'Angelo and Casali, 2012). For instance, transgenic mice expressing GFP under the glycine transporter 2 (GlyT2) promoter are used to identify GoCs, yet GFP-positive neurons in these mice include approximately 85% of GoCs and other types of neurons, but exclude 15% of GoCs that are purely GABAergic (Simat et al., 2007b). However, all GoCs have two classes of dendrites, basal and apical, and a widely ramified axon (Barmack and Yakhnitsa, 2008). Basic circuit structures also appear to be conserved among the GoCs, which provide feedforward and feedback inhibition onto GCs (Figure 4A-a) through excitatory synaptic connections from MFs to basal dendrites in the GL (Figure 4A-b) and from PFs to apical dendrites in the ML (Figure 4A-c), respectively. The regulation mediated by GoCs appears to be critical for fine-tuning GC activity (Crowley et al., 2009; Fleming et al., 2024).

In addition to these basic circuit structures, several studies have demonstrated additional inputs onto GoCs. While excitatory inputs from GCs are predominantly made through PFs in the ML, it has also been shown that ascending axons of GCs form functional synaptic contacts onto the proximal dendrites of GoCs in the GL (Figure 4B; Cesana et al., 2013). Although early studies suggested synaptic contacts from the CFs to GoCs (Scheibel and Scheibel, 1954; Marr, 1969), subsequent research did not find such contacts (Galliano et al., 2013). Another study demonstrated that CFs have excitatory effects on GoCs through spillover-mediated transmission, similar to the case with MLIs (Figure 4C; Nietz et al., 2017). In contrast to the clear excitatory synaptic inputs from MFs and PFs onto GoCs, the source of inhibitory synaptic inputs remained unidentified for a long time and is still not completely clear (Figure 4D). It was suggested that LuCs provide inhibitory inputs via the release of glycine and GABA in the presence of serotonin, and MLIs also provide GABAergic inhibitory inputs (Dumoulin et al., 2001). However, studies using paired patch-clamp recordings or anatomical analyses demonstrated that GoCs do not receive inhibitory inputs from MLIs (Hull and Regehr, 2012; Eyre and Nusser, 2016). Instead, functional synaptic responses between neighboring GoCs were detected in 20% of all connections tested (Hull and Regehr, 2012). Considering the anatomical observation that a small fraction of GABAergic synaptic inputs to GoC dendrites contain GlyT2, a marker of glycinergic terminals (Eyre and Nusser, 2016), it is suggested that LuCs, which release both GABA and glycine, may actually innervate GoCs. Alternatively, some CN neurons, which also release a mixture of GABA and glycine, have been shown to innervate GoCs (Ankri et al., 2015).

GoCs can be subdivided based on their heterogeneous properties, which may have biological implications. Studies have attempted to classify GoC subtypes based on biochemical and neurochemical properties (Geurts et al., 2001; Simat et al., 2007b), such as the expression of Rat-303, metabotropic glutamate receptor (mGluR) 2, somatostatin, and neurogranin in glycinergic and/or GABAergic neurons. Although the direct association between the biochemical subtypes of GoCs and their electrophysiological properties has not yet been clarified, synaptic connections that are selective or preferable for specific GoC subtypes have been reported (Ankri et al., 2015; Eyre and Nusser, 2016). GlyT2-positive inhibitory axons preferentially make synapses onto neurogranin-positive and GlyT2-negative GoCs rather than neurogranin-negative and GlyT2-positive GoCs (Eyre and Nusser, 2016). Consistently, CN neurons releasing both GABA and glycine selectively, but not exclusively, innervate purely GABAergic GoCs (Ankri et al., 2015), which are neurogranin-positive and GlyT2-negative (Simat et al., 2007b). Thus, there may be GoC subtype-dependent circuit organizations, suggesting functional differences among the biochemical and neurochemical subtypes of GoCs.

Similar to the MLIs, GoCs are also electrically coupled via gap junctions (Figure 4E; Dugué et al., 2009; Vervaeke et al., 2010; Hull and Regehr, 2012). Both depolarization and hyperpolarization can be transmitted through these gap junctions, but the inhibition of neighboring GoCs by the transmission of spike afterhyperpolarization appears to be a major effect of their electrical coupling (Dugué et al., 2009; Vervaeke et al., 2010). Computational models suggest that the electrical coupling of GoCs with excitatory inputs from MFs regulates the synchronization and desynchronization of GoC population activity (Dugué et al., 2009; Vervaeke et al., 2010). This dynamic regulation of population activity in local GoC circuits was indeed observed in awake animals (Gurnani and Silver, 2021). These findings raise the possibility that electrical coupling and excitatory synaptic inputs may be the major regulators of GoCs, while relatively minor inhibitory synaptic inputs may modulate specific pathways.

## 6 Circuits of traditionally minor neuron types

In addition to the major neurons described above, at least four other types of neurons have been reported in the cerebellar cortex (Schilling et al., 2008). Although the cerebellum is a well-studied brain region, these other neuron types are less well understood. Presumably owing to the very small numbers of them within the cerebellum, which is densely packed with GCs and PCs, investigating these minor neurons has been difficult. The lack of clearly distinguishable molecular markers also poses a challenge (Tam et al., 2021). Consequently, there is no unified categorization of these neuron types (Geurts et al., 2001; Kozareva et al., 2021; Tam et al., 2021). Nevertheless, the basic anatomical properties of these relatively minor neuron types are gradually being clarified, and their functions have been proposed based on their anatomical properties.

### 6.1 UBCs

UBCs are the only excitatory interneurons among them and are located in the GL of the cerebellar vermis. Specifically, UBCs are abundant in cerebellar regions associated with vestibular functions, such as lobules IX and X in the vermis, but are relatively rare in other lobules (Diño et al., 1999). They have a distinctive morphology, characterized by a single short dendrite with a brush-like end. UBCs receive an excitatory input from a single MF and inhibitory inputs from GoCs (Figure 4F-a; Harris et al., 1993; Floris et al., 1994; Kalinichenko and Okhotin, 2005; Mugnaini et al., 2011; McDonough et al., 2020). Their axons branch out locally and form MF terminal-like structures in the GL, which make synapses onto GCs or other UBCs (Mugnaini et al., 2011; van Dorp and De Zeeuw, 2015). UBCs have been thought to temporally amplify short-lived signals arising from a single MF by utilizing their unique circuit structures (Kinney et al., 1997; Mugnaini et al., 2011; van Dorp and De Zeeuw, 2014). However, several studies suggested that UBCs have more complex circuits and functions. Two biochemical subtypes of UBCs have been identified: mGluR1α-positive UBCs, which lack calretinin, and mGluR1α-negative UBCs, which express calretinin (Nunzi et al., 2002; Diño and Mugnaini, 2008). Functionally distinct subtypes appear to be associated with these biochemical differences: MF inputs excite mGluR1α-positive UBCs, but suppress firing in mGluR1α-negative UBCs (Russo et al., 2008; Borges-Merjane and Trussell, 2015; Zampini et al., 2016). Subtype-specific circuit structures have also been reported (Figure 4F-b). Primary sensory afferents from the vestibular organ selectively innervate mGluR1α-positive UBCs, while secondary sensory afferents from the vestibular nucleus innervate both UBC subtypes (Balmer and Trussell, 2019). Additionally, inhibitory connections from PC collateral axons specifically innervate mGluR1α-positive UBCs (Guo et al., 2021). Given these specific circuit structures, UBCs may mediate bidirectional activity patterns in distinct GC pathways by amplifying excitatory signals in one pathway while suppressing firing in another.

### 6.2 LuCs

LuCs are a type of inhibitory interneuron in the GL, characterized by fusiform somas located beneath PC somas, and are intermediate in size between GoCs and globular cells, which are two other inhibitory interneurons in the GL. Anatomical studies demonstrated that LuCs extend poorly ramified horizontal and bipolar dendrites beneath the PC somas, where they receive inhibitory inputs from PC collateral axons, and project their axons to the ML, where they innervate MLIs (Lainé and Axelrad, 1996, 1998; Simat et al., 2007b). As mentioned above, the functional synaptic connections from PCs to LuCs were confirmed by electrophysiological recordings upon optogenetic activation of PCs (Witter et al., 2016). Another recent study presented a wider range of anatomical connections, revealing inputs from CFs, MFs, GC ascending axons, GoCs, other LuCs, and serotonergic fibers, and axonal projections to GoC dendrites (Figure 4G), but not to PC dendrites (Miyazaki et al., 2021). This raises an intriguing possibility that LuCs integrate various inputs and then coordinate two types of inhibitory circuits, though further functional studies are necessary (Miyazaki et al., 2021).

### 6.3 Globular cells

Globular cells have often been considered a subtype of LuCs (Lainé and Axelrad, 2002; Schilling et al., 2008; Prestori et al., 2019). Although globular cells exhibit different morphological properties, such as having a small and round soma with radiating dendrites, they share circuit properties with LuCs, in terms of inputs from PC collateral axons and projections to the ML (Lainé and Axelrad, 2002). One functional difference reported was that globular cells responded to both serotonin and noradrenaline, whereas small LuCs responded only to serotonin (Hirono et al., 2012). In addition, studies using single-cell transcriptomic analysis suggested three types of inhibitory interneurons around the PCL, presumably LuCs, globular cells, and candelabrum cells (Kozareva et al., 2021; Osorno et al., 2022). Although the functional relevance of distinguishing LuCs and globular cells is still unclear, the possibility is gradually emerging that they exhibit unique functions due to their distinct molecular expressions.

### 6.4 Candelabrum cells

Candelabrum cells were identified more than two decades ago as neurons with a small soma situated in the PCL, with a few dendrites extending toward the surface of the ML and axons winding through or above the PCL (Lainé and Axelrad, 1994). They have long been the most enigmatic cell type in the cerebellum. Interestingly, a recent study established a mouse line showing fluorescent labeling in candelabrum cells, sufficiently distinguishing them from other cell types (Osorno et al., 2022). The study found that GABAergic candelabrum cells receive excitatory inputs from MFs and GCs, and strong inhibitory inputs from PC collateral axons, while they inhibited MLIs (Figure 4H). This mouse line may thus facilitate in unraveling the long-standing mystery of candelabrum cells.

## 7 Discussion: Structural properties that need further clarification

As we have described throughout this article, new structural properties have been demonstrated in the cerebellar cortex. Although the cerebellum has long been thought to consist of an orderly repetition of simple structures, recent findings have revealed that it is actually more complex than previously expected. However, the more details we learn, the more questions arise. Since it is now generally accepted that the cerebellum is involved in cognitive and affective functions in addition to motor functions, there will likely be more studies addressing how the cerebellum processes multiple types of information. For such studies, it is crucial to further understand cerebellar circuit properties. Here, we discuss circuit structures that should be elucidated by considering newly identified structural properties.

### 7.1 Feedforward and feedback microcircuits

**Figure 5:**
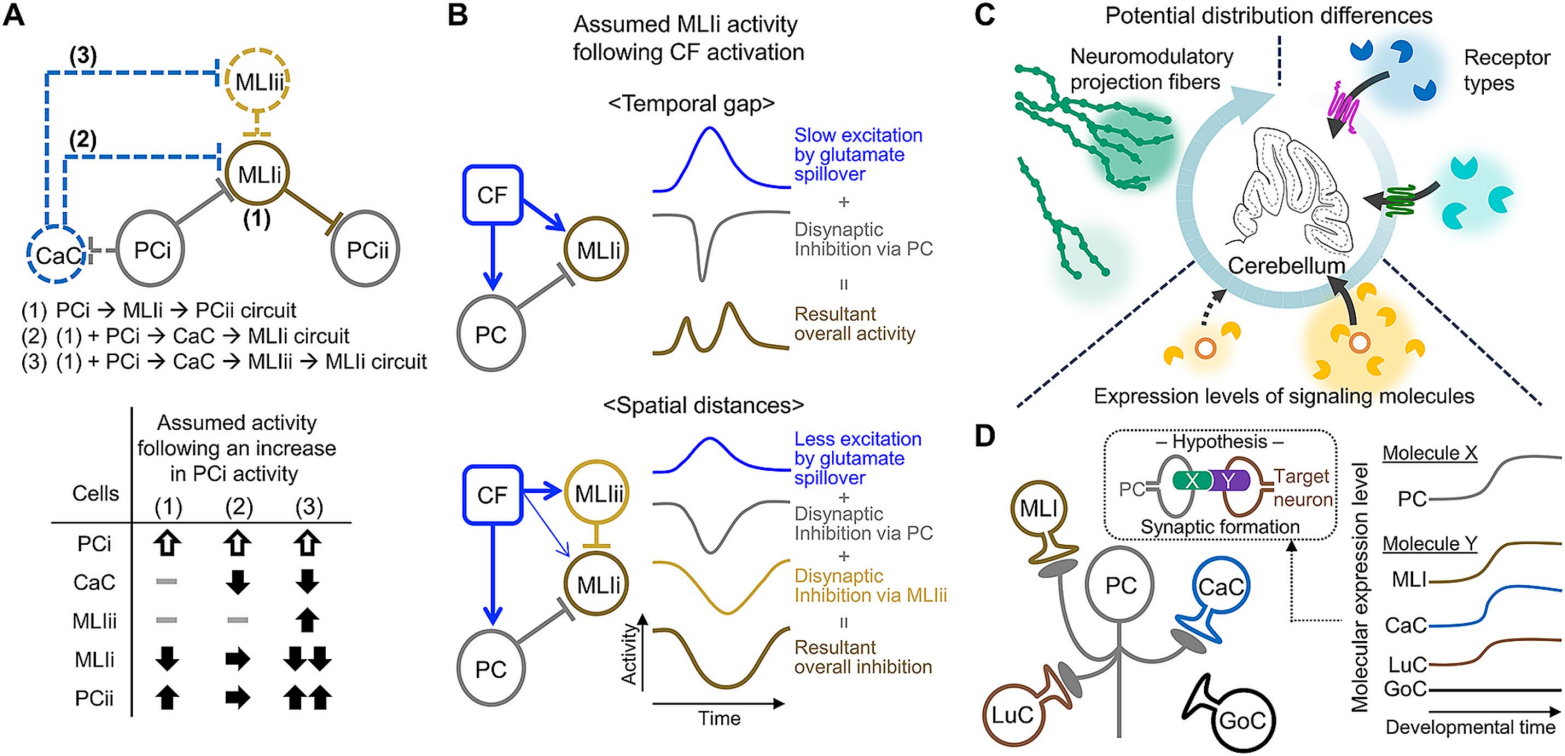
Examples of structural properties requiring further clarification. **(A)** Three possible circuit arrangements among PCs, MLIs, and candelabrum cells (CaCs) at the individual neuron level (top). The activities of neurons in these circuits may differ in response to an increase in PCi activity (bottom). **(B)** Two examples of circuit structures involving MLIi, which receives conflicting inputs: excitation from glutamate spillover originating from CF and PC-dependent inhibition. The time course of MLIi activity following CF activation may vary depending on whether the excitatory and inhibitory inputs are temporally distinct (top) or spatially organized (bottom). **(C)** Potential mechanisms of other factors (e.g., neuromodulators or circulating molecules) exhibiting spatial selectivity. **(D)** A diagram illustrating the use of developmental time-and cell type-dependent transcriptome analysis data in the research on mechanisms of specific synaptic formation of PC axon collaterals. If the expression of hypothetical molecules X and Y increases in PCs and target neurons, respectively, during developmental periods, these molecules may play a role in synaptic formation. Note that the Roman numbers following PC or MLI in this figure (e.g., PCi or MLIi) are used to differentiate between distinct PCs or MLIs.

Feedforward and feedback microcircuits are fundamental components of neural computations, widely distributed across various brain regions (D'Souza and Burkhalter, 2017; Park et al., 2023). Among these regions, the cerebellum stands out: GCs receive both feedforward and feedback inhibitions from GoCs, and PCs receive feedforward inhibition from MLIs (D'Angelo et al., 2013; Kim and Augustine, 2021). Furthermore, studies have revealed additional microcircuits within the cerebellar cortex, including feedback inhibitions on various neuron types from PCs and feedforward excitation on GCs mediated by UBCs (Kinney et al., 1997; Mugnaini et al., 2011; van Dorp and De Zeeuw, 2015; Guo et al., 2016; Witter et al., 2016; Guo et al., 2021; Osorno et al., 2022; Hariani et al., 2024). Although the specific arrangements of these microcircuits have been gradually elucidated (Balmer and Trussell, 2019; Guo et al., 2021; Halverson et al., 2022), a comprehensive understanding remains elusive. For example, PC collateral axons send feedback inhibitions to GCs, UBCs, LuCs, candelabrum cells, and MLIs, some of which connect each other, yet their circuit arrangements at the individual neuron level are not clear. There might be organized connections among them, similar to the connections between MLIs and PCs: MLIs appear to receive feedback inhibition from PCs that are distinct from the PCs innervated by those particular MLIs (Halverson et al., 2022). Since the resulting neuronal activity and potential regulations can differ according to the circuit arrangements (Figure 5A), further analyses utilizing advanced techniques, such as a combination of sparse labeling and anterograde transsynaptic labeling, are expected.

### 7.2 Superficially conflicting functions among new structural organization

A large amount of effort has been made over the past decade to advance our understanding of cerebellar circuits. We assume that the additional organization of cerebellar circuits increases the options of regulating PC activity, consequently leading to pattern separations of signals provided by the cerebellum. However, it is still difficult to picture exactly how such circuit organization collaboratively controls PC activity. Superficially contradicting aspects make it even more challenging. For example, in MLI circuits, MLIs in the inner ML or MLI1s likely have strong inhibitory effects on PCs due to their high efficiency of individual inhibitory synaptic inputs (Arlt and Häusser, 2020) and strong electrical coupling (Alcami and Marty, 2013; Lackey et al., 2024). However, they are inhibited by MLIs in the outer ML or MLI2s (Rieubland et al., 2014; Lackey et al., 2024). This raises concerns that the strong inhibitory effects from the inner MLIs might be underutilized or wasted. Additionally, glutamate spillover from CFs (Coddington et al., 2013; Arlt and Häusser, 2020) and feedback signals from PCs (Witter et al., 2016) could have both excitatory and inhibitory effects on MLIs, raising the possibility that they might functionally cancel each other out. These concerns might be alleviated by obtaining more precise information about the spatiotemporal aspects of MLI circuits, such as temporal gaps or the distinct spatial distances between excitatory and inhibitory effects (Figure 5B). A study provided a clue about the first concern by recording the activity of MLI1s, MLI2s, and PCs during licking, which periodically modulates PC firing (Lackey et al., 2024). Changes in PC firing rates were inversely correlated with MLI1s and correlated with MLI2s, suggesting the organization of bidirectional control of PC firing by these two groups of MLIs. Alternatively, it would be interesting to computationally predict the tuning of PC activities by including all known components of the complex MLI circuit organizations.

### 7.3 Other factors involved in the cerebellar circuit structures

While this article focuses on neurons and excitatory inputs in the cerebellar cortex, many other factors also contribute to cerebellar circuit structures as components or regulators. These include non-neuronal cells, neuromodulators, and molecules arriving from circulation (Schweighofer et al., 2004; Koibuchi and Ikeda, 2013; Araujo et al., 2019; Guedes et al., 2022; Gruol, 2023; Li et al., 2024). To comprehensively understand cerebellar circuit structures and their functions, these factors must also be taken into account. Indeed, information on their contributions has gradually been updated. For example, immunohistochemical analyses have investigated layer-or lobule-dependent distributions and orientations of neuromodulatory projection fibers (Carlson et al., 2021; Longley et al., 2021). Other studies have reported novel interactions between PCs and Bergmann glial cells through dopamine released from PCs (Li et al., 2023), and cerebellar circuit formation through cytokine-dependent regulation of microglial functions (Kana et al., 2019; Guedes et al., 2023). Regulation by these factors appears less selective than synaptic transmission-mediated regulation, as evidenced by volume transmission of neuromodulators (Özçete et al., 2024) and delivery of cytokines through circulation (Wu et al., 2023). Nevertheless, recent studies have shown their involvement in specific cerebellar functions or disfunctions (Yamamoto et al., 2019; Kim et al., 2021; Cutando et al., 2022; Snell et al., 2022; Guedes et al., 2023; Li et al., 2023; Stanley et al., 2023; Dewa et al., 2024). As the broad range of cerebellar functions appear to be achieved through computations in different functional modules/domains, as shown in human studies (Guell et al., 2018; Diedrichsen et al., 2019; King et al., 2019), it would be intriguing to determine how the factors described in this paragraph exhibit a certain extent of spatial selectivity within the cerebellum (Figure 5C).

### 7.4 Circuit organization during postnatal development

Well-organized circuit structures of the cerebellum gradually form during postnatal development through dynamic processes regulated by both cell-autonomous mechanisms and interactions between developing neurons in the cerebellum (Leto et al., 2016; Park et al., 2021; van der Heijden and Sillitoe, 2021; Kim et al., 2023a). While extensive research has focused on these processes for traditionally known circuit structures, less is understood about the developmental mechanisms of newly identified structures. The updated structures are relatively minor or complex, complicating the investigation of their developmental processes. In general, a useful approach to understand developmental processes may be to examine the effects of molecular deficiencies during specific developmental windows. Developmental time-and cell type-dependent transcriptome analysis data could help to identify relevant molecules (Figure 5D). This approach indeed led us to discover developmental mechanisms that maintain the PC soma monolayer after its formation. Specifically, multiple epidermal growth factor-like domains protein 11, which substantially increases during the late stage of GC development (Rosenberg et al., 2018), plays a role in this maintenance (Jun et al., 2023). Furthermore, integrating transcriptomic data with known information about circuit formation could be beneficial for hypothesizing the developmental mechanisms of the updated structures. For example, neuropilin-1 (Nrp1) expressed in BCs is crucial for the formation of pinceau synapses in the AIS of PCs (Telley et al., 2016). Whereas candelabrum cells also express high levels of Nrp1, they do not innervate PCs (Osorno et al., 2022). Two potential mechanisms are proposed to explain this discrepancy (Schilling, 2024): the timing of expression of Nrp1 and its partner proteins in PCs, MLIs, or candelabrum cells might be regulated, or different splice variants of Nrp1 may contribute differently to their synaptic connections. Thus, hypotheses based on existing data would help advance our understanding of how cerebellar circuits, including newly identified structures, are coordinately organized during postnatal development.

## 8 Conclusion and perspectives

This article highlights recent advances in our understanding of cerebellar cortex circuit structures, including greater heterogeneity among neuron types, previously unidentified synaptic connections, and more complex circuit organizations. In general, characteristic circuit structures within the cerebellum are believed essential for neural computations and their roles in behavior (D'Angelo et al., 2011; Billings et al., 2014). In line with this belief, existing theories propose functions for some newly identified circuit structures, such as aspects of MLI organization that may contribute to the functional microdomains of PCs (Coddington et al., 2013; Witter et al., 2016; Hoehne et al., 2020; Halverson et al., 2022), or the two types of UBCs that may convey different vestibular information bidirectionally to distinct GC pathways (Russo et al., 2008; Borges-Merjane and Trussell, 2015; Zampini et al., 2016; Balmer and Trussell, 2019). Ideally, our understanding of the structural-functional relationships in cerebellar circuits will be enhanced through the proposal and validation of further theories. Constructing theoretical models based on experimental observations would be valuable for comprehensively predicting the cooperative or antagonistic effects of various circuit structures. The development of techniques for targeted manipulations, such as inhibiting specific connections, collateral axons, or cell types, would facilitate experimental validation. Transcriptomic data could be instrumental in this technical development. It is crucial to investigate through these analyses how the unique circuit structures and neuronal morphologies in the cerebellar cortex contribute to neural computation, extending beyond the structural studies discussed in the previous section (6.1). This would advance our understanding of cerebellar functions and the broader mechanisms underlying brain operation.
